# Management of lumbar spondylodiscitis developing after laparoscopic sacrohysteropexy with a mesh: A case report and review of the literature

**DOI:** 10.1097/MD.0000000000018252

**Published:** 2019-12-10

**Authors:** Da-Cheng Qu, Hong-Bin Chen, Mao-Mei Yang, Hong-Gui Zhou

**Affiliations:** Department of Obstetrics and Gynecology, Affiliated Hospital of North Sichuan Medical College, Nanchong, China.

**Keywords:** lumbar spondylodiscitis, sacral colpopexy, sacral rectopexy, sacrohysteropexy

## Abstract

**Introduction:**

Lumbar spondylodiscitis is a rare and severe complication of laparoscopic sacrohysteropexy with a polypropylene mesh. However, a case of lumbar spondylodiscitis following laparoscopic sacrohysteropexy has not been reported so far. We present a case of lumbar spondylodiscitis following laparoscopic sacrohysteropexy with a mesh. We also discuss 33 cases of lumbar spondylodiscitis following sacral colpopexy and (or) rectopexy with a mesh.

**Patient concerns:**

A 46-year-old woman with 3 previous vaginal deliveries underwent laparoscopic mesh sacrohysteropexy for stage III uterine prolapse. One month after surgery, the patient developed persistent symptoms, such as stiffness of the lumbosacral portion, low back pain (LBP), persistent swelling, pain between the right iliac crest and the buttock, inability to bend down, and pain in the right lower limb. Symptoms were alleviated by a nonsteroidal anti-inflammatory drug. However, in the last 7 days, symptoms worsened and she was unable to stand or walk. The patient had very limited leg mobility.

**Diagnosis:**

Blood routine examination, erythrocyte sedimentation rate, C-reactive protein, and magnetic resonance imaging (MRI) of the lumbar spine indicated lumbar pyogenic spondylodiscitis.

**Interventions:**

Removal of mesh and hysterectomy via laparoscopy were performed immediately, and antibiotics were given simultaneously. However, on the basis of MRI findings and persistent symptoms, debridement, laminectomy, spinal canal decompression, bone grafting, and internal fixation via pedicle screw placement were performed 5 months after laparoscopic sacrohysteropexy.

**Outcomes:**

All symptoms were alleviated 5 days after the operation. The patient could stand in the erect position and raise her lower limbs within 2 weeks. She could resume her normal activities within 2 months after the operation, and her X-ray appeared normal.

**Conclusion:**

Persistent LBP and radiating pain may be the signals of lumbar spondylodiscitis. MRI is the gold standard diagnostic examination for lumbar spondylodiscitis. Awareness of symptoms, such as LBP and radiating pain symptoms, timely diagnosis, mesh removal, and referral to orthopedists are important to prevent more severe complications. Surgical practice needs to be improved further and any other infections should be treated immediately as the most likely causes of lumbar spondylodiscitis are related to the mesh and other infections.

## 1 Introduction

Laparoscopic sacrohysteropexy with a mesh is a variation of the sacral colpopexy to correct apical prolapse in women who desire uterine preservation.^[1]^

Spondylodiscitis, also referred to as pyogenic discitis and vertebral osteomyelitis, is defined as an infection limited to the intervertebral disc (discitis) and the adjacent vertebrae (vertebral osteomyelitis).^[2]^ Spondylodiscitis is a condition that includes a spectrum of spinal infections such as discitis, osteomyelitis, epidural abscess, meningitis, subdural empyema, and spinal cord abscess.^[3]^ Awareness of symptoms, timely diagnosis, and multidisciplinary approach to the management of this condition are important to prevent other severe complications.^[4]^

We present a case of lumbar spondylodiscitis following laparoscopic sacrohysteropexy with a mesh that was referred to orthopedists for surgery. We also evaluate the current literature to understand how to manage lumbar spondylodiscitis developing after laparoscopic sacrohysteropexy with a mesh.

## 2 Case report

**Figure 1:**
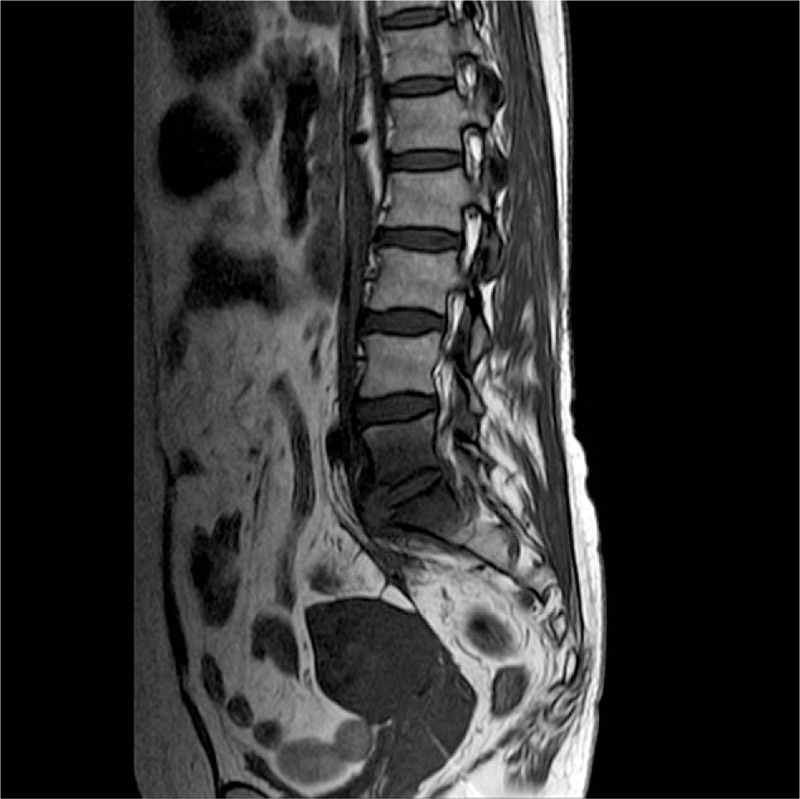
MRI: Lumbar pyogenic spondylodiscitis, enhancement of soft tissues surrounding the L5-S1 vertebrae (arrow). MRI = magnetic resonance imaging.

**Figure 2:**
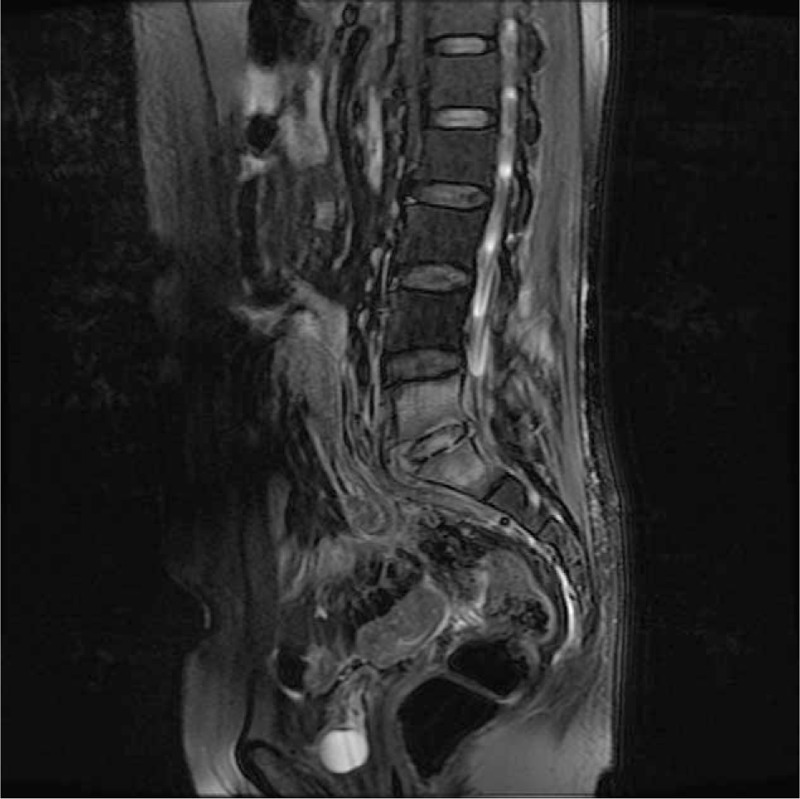
MRI after mesh removal 2 wk later: bony destruction of the lower part of the L5 vertebra and the dome of the sacrum (arrow). MRI = magnetic resonance imaging.

**Figure 3:**
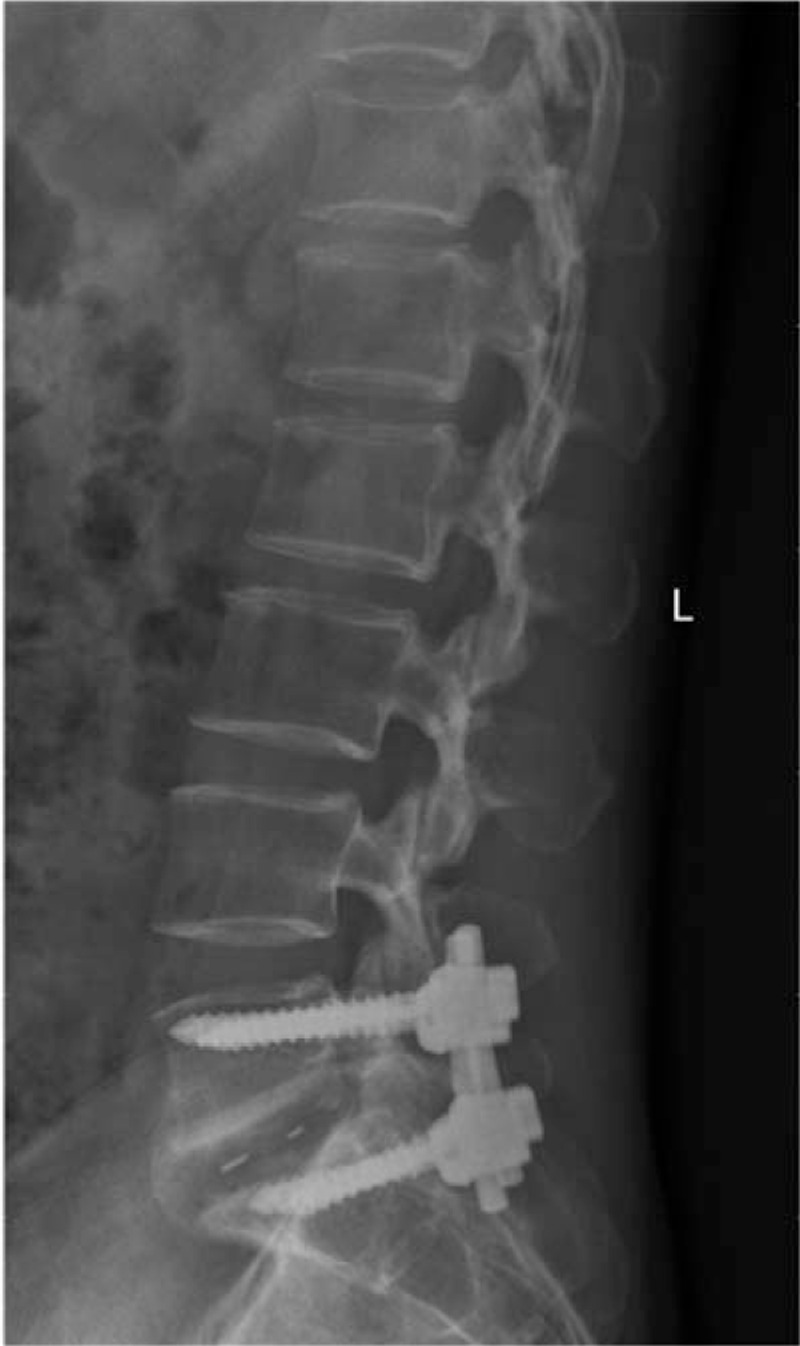
Lumbar vertebral X-ray: normal findings (arrow).

A 46-year-old woman with 3 previous vaginal deliveries suffered from stage III uterine prolapse for 1 year and it had worsened during the last 6 months. Five months ago, laparoscopic sacrohysteropexy was performed using a Y-shaped polypropylene mesh. A prophylactic antibiotic was used within 24 hours in the absence of infection. Her past medical history was unremarkable. After the procedure, the pelvic organ prolapse disappeared. One month after surgery, the patient developed discomfort in the lumbosacral portion that continuously persisted for 4 months. The symptoms included stiffness of the lumbosacral portion, low back pain (LBP), persistent swelling, pain between the right iliac crest and the buttock, inability to bend down, and pain in the right lower limb. Symptoms were alleviated after a nonsteroidal anti-inflammatory drug was prescribed in the outpatient clinic. But in the last 7 days, symptoms were not alleviated and they worsened, and the patient was unable to stand or walk. The legs could only be moved away from the bed up to 20 cm because of pain. Primitive reflexes were negative and there was no loss of sensory function. Then the patient was referred for admission. Blood routine examination, erythrocyte sedimentation rate, C-reactive protein, and magnetic resonance imaging (MRI) of the lumbar spine indicated lumbar pyogenic spondylodiscitis (Fig. 1). Y-shaped polypropylene mesh was removed and hysterectomy was conducted via laparoscopy immediately, and antibiotics were given simultaneously. During the operation, a festering wound was seen at the location of the stitches over the lumbosacral portion and the mesh suture was placed higher than its usual level. *Escherichia coli* bacteria were found at the location of the stitches. Tienam was given for 2 weeks as *E coli* bacteria are sensitive to this drug. The patient was referred to orthopedists because of persistent symptoms. MRI indicated bony destruction of the lower part of the L5 vertebra and the dome of the sacrum and absence of favorable evolution (Fig. 2). Through the retroperitoneal lumbar approach, destruction of the vertebral body between the fifth lumbar vertebra and the first sacral vertebra was seen, and degeneration of the intervertebral disc and necrosis of the lumina of the L5 vertebra were also seen. Debridement, laminectomy, spinal canal decompression, bone grafting, and internal fixation via pedicle screw placement were conducted 5 months after laparoscopic sacrohysteropexy. Symptoms were alleviated 5 days after the operation, and the pain decreased. The patient was discharged on the seventh day after the operation. The patient was able to stand in the erect position and raise her lower limbs within 2 weeks. The patient returned to normal activity within 2 months after the operation, and the X-ray also appeared normal (Fig. 3).

## 3 Discussion

Laparoscopic sacrohysteropexy with a mesh is considered to be the gold standard for apical prolapse in young women who desire uterine preservation.^[1]^ Mesh graft can lead to infections and graft rejection, and different conditions require different management therapies.^[5]^ Lumbar spondylodiscitis is a rare and severe complication, which can cause LBP, fever, and radiating pain symptoms, such as pain in the buttock and leg, and even mobility limitation.^[6]^ A case of lumbar spondylodiscitis following laparoscopic sacrohysteropexy has not been reported so far. Only cases of lumbar spondylodiscitis following sacral colpopexy and (or) rectopexy with a mesh have been reported.

We performed a literature search via PubMed, and we found 33 cases of lumbar spondylodiscitis following sacral colpopexy and (or) rectopexy with a mesh (Table 1  ). Summary of the characteristics is presented in Table 2. Thirty-four women with a median age of 60 years (range, 42–80 years) were diagnosed with spondylodiscitis following sacrohysteropexy, sacral colpopexy, and (or) rectopexy with a mesh. They visited their doctors with symptoms. The median time to symptom presentation was 14 months, and it ranged from 6 days to 8 years. LBP occurred in every case. Further, 38% of the patients suffered from fever and 35% of the patients suffered from radiating pain symptoms, which mostly predicted the need for referral to orthopedists for surgical interventions (Table 3). Regardless of the recovery time after surgery, the possibility of spondylodiscitis must be considered when the patients present with LBP, fever, and especially radiating pain symptoms, which may predict the presence of severe spondylodiscitis requiring multidisciplinary surgical interventions.

**Table 1:**
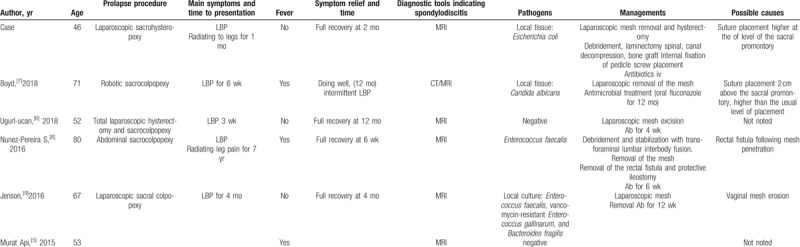
Cases of spondylodiscitis following sacrohysteropexy, sacral colpopexy, and rectopexy with a mesh.

**Table 1 (Continued):**
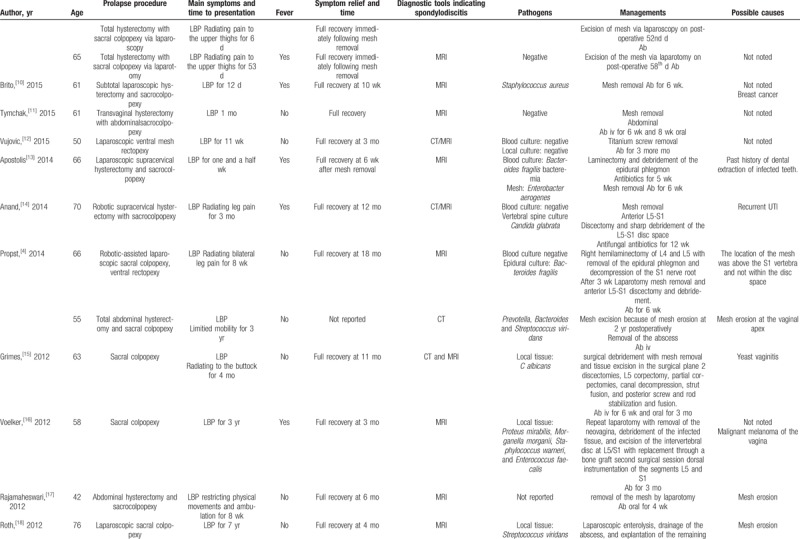
Cases of spondylodiscitis following sacrohysteropexy, sacral colpopexy, and rectopexy with a mesh.

**Table 1 (Continued):**
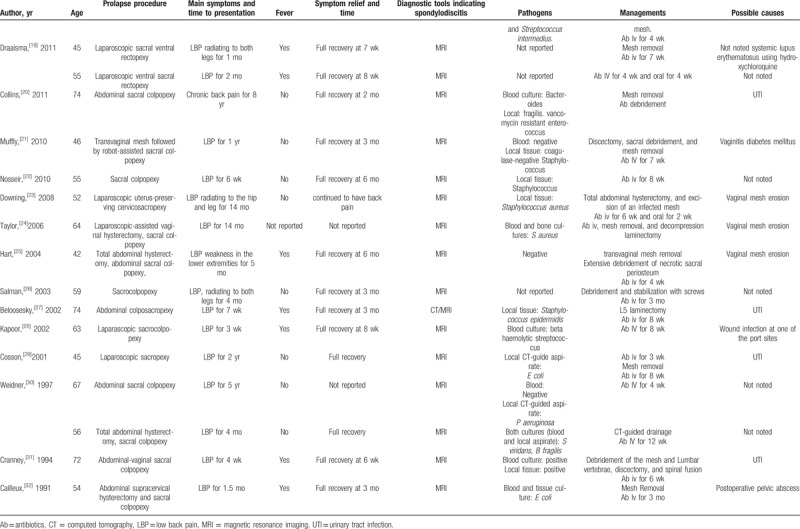
Cases of spondylodiscitis following sacrohysteropexy, sacral colpopexy, and rectopexy with a mesh.

**Table 2:**
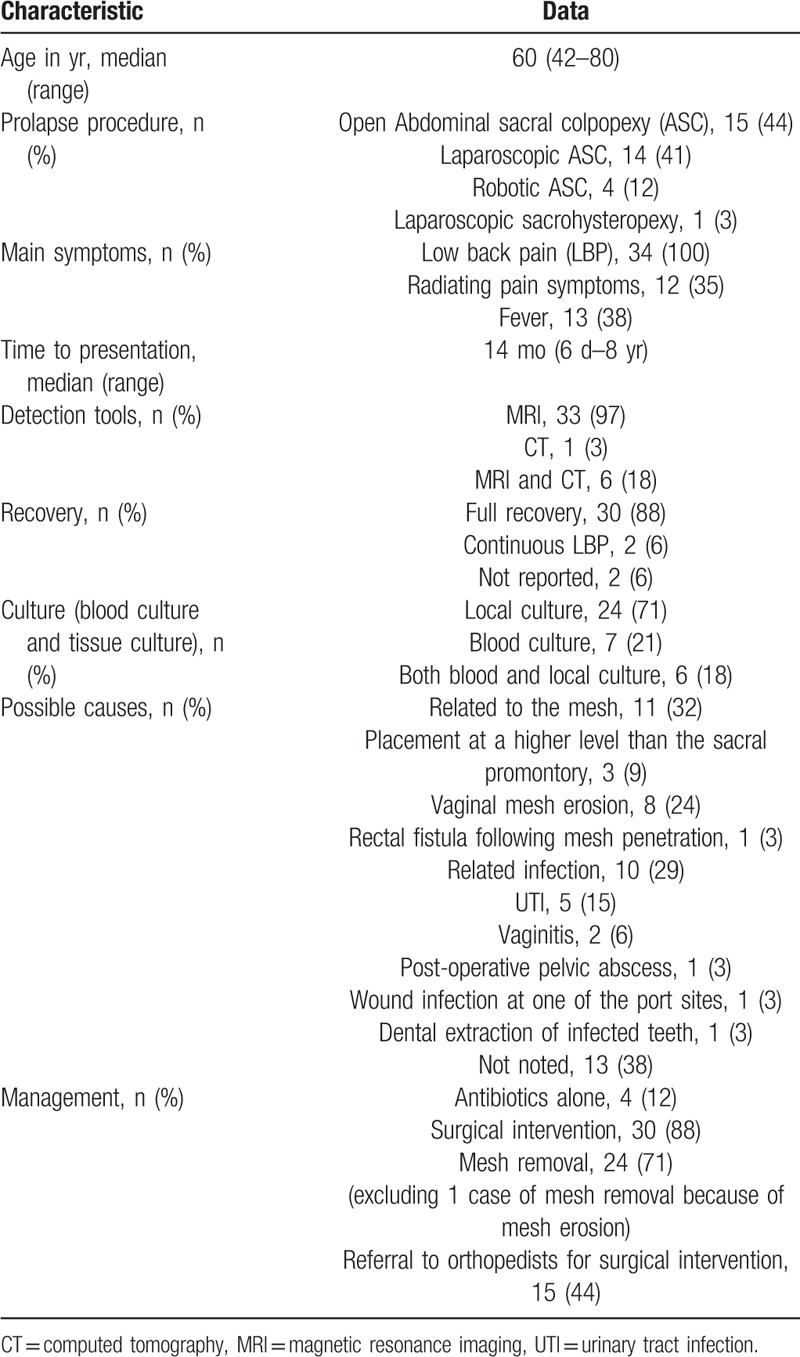
Summary of the characteristics of cases of spondylodiscitis following sacrohysteropexy, sacral colpopexy, and rectopexy with a mesh (including the presented case and 33 cases from literature review).

**Table 3:**
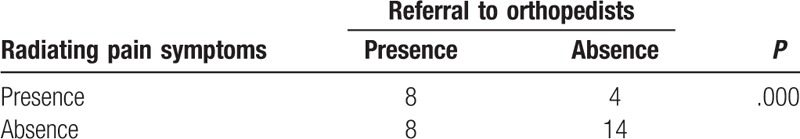
The relationship between occurrence of radiating pain symptoms and referral to orthopedists.

MRI can indicate the early stage of spondylodiscitis reflecting the obvious imaging changes. In our study, 97% of cases of spondylodiscitis were diagnosed by MRI; excluding 1 case that was diagnosed by computed tomography (CT) and MRI was not conducted. CT-guided aspiration can sometimes be useful for diagnosing local infection.

Seventy-one percent of the patients developed local pathogen infection, which included bacterial and fungal infections. It was only infection, and not graft rejection.^[5]^ CT-guided aspiration can sometimes be useful for diagnosing local infection and for prescribing the correct drugs.

Antibiotics alone were effective in only 4 cases, that is, 12% of the total cases; thus, showing a low percentage. Most of the patients needed surgical interventions. Mesh removal and debridement were effective in majority of the cases, while 44% of the cases needed multidisciplinary surgical interventions, mainly orthopedic surgery. Further, 88% of the patients were able to return to normal daily activity, and only 6% of the patients suffered from intermittent LBP.

The possible causes of lumbar spondylodiscitis were mainly related to the mesh (32%) and other infections (29%), while the other causes of lumbar spondylodiscitis were not noted. Mesh-related causes included vaginal mesh erosion (24%), including mesh penetration into the rectum in 1 case, and suture placement at a level higher than the usual placement level (9%). Other infections included urinary tract infection in 5 cases (15%), vaginitis in 2 cases (6%), postoperative pelvic abscess in 1 case (3%), wound infection at 1 of the port sites in 1 case (3%), and dental extraction of infected teeth in 1 case (3%). Surgical practice needs to be improved further and any other infections should be treated immediately.

In conclusion, persistent LBP and radiating pain may be the signals of lumbar spondylodiscitis. MRI is the gold standard diagnostic examination for lumbar spondylodiscitis. Awareness of symptoms, especially LBP, fever, and radiating pain symptoms, and timely MRI of the lumbar spine can help the early diagnosis of spondylodiscitis. Local bacterial culture can be useful for prescribing more effective antibiotics. Mesh removal and debridement are the main gynecological surgical interventions. Timely referral to orthopedists can prevent additional surgeries.

## Author contributions

**Conceptualization:** Da-Cheng Qu, Hong-Bin Chen, Hong-Gui Zhou.

**Data curation:** Da-Cheng Qu.

**Formal analysis:** Da-Cheng Qu, Hong-Bin Chen.

**Funding acquisition:** Mao-Mei Yang.

**Investigation:** Da-Cheng Qu, Mao-Mei Yang.

**Methodology:** Hong-Bin Chen, Mao-Mei Yang.

**Project administration:** Hong-Bin Chen, Hong-Gui Zhou.

**Software:** Da-Cheng Qu.

**Supervision:** Hong-Gui Zhou.

**Validation:** Hong-Gui Zhou.

## References

[1] Betschart C Cervigni M Contreras Ortiz O Management of apical compartment prolapse (uterine and vault prolapse): a FIGO Working Group report. Neurourol Urodyn 2017;36:507–13. 26485226 10.1002/nau.22916

[2] Rosinsky P Mandler S Netzer N Antibiotic-resistant spondylodiscitis with canal invasion and aggressive evolution to epidural abscess: a case series of spontaneous occurrence in 16 patients. Int J Spine Surg 2018;12:743–50. 30619679 10.14444/5093 PMC6314345

[3] Cottle L Riordan T Infectious spondylodiscitis. J Infect 2008;56:401–12. 18442854 10.1016/j.jinf.2008.02.005

[4] Propst K Tunitsky-Bitton E Schimpf MO Pyogenic spondylodiscitis associated with sacral colpopexy and rectopexy: report of two cases and evaluation of the literature. Int Urogynecol J 2014;25:21–31. 23775373 10.1007/s00192-013-2138-3

[5] Api M Kayatas S Boza A Spondylodiscitis following sacral colpopexy procedure: is it an infection or graft rejection? Eur J Obstet Gynecol Reprod Biol 2015;194:43–8. 26321411 10.1016/j.ejogrb.2015.08.003

[6] Gungor Ugurlucan F Yasa C Demir O Long-term follow-up of a patient with spondylodiscitis after laparoscopic sacrocolpopexy: an unusual complication with a review of the literature. Urol Int 2018;1–5. 10.1159/000494370 30485841

[7] Boyd B Pratt T Mishra K Fungal lumbosacral osteomyelitis after robotic-assisted laparoscopic sacrocolpopexy. Female Pelvic Med Reconstr Surg 2018;24:e46–8. 30059439 10.1097/SPV.0000000000000612

[8] Nunez-Pereira S Huhmann NV Rheinwalt KP Lumbosacral spondylodiscitis due to rectal fistula following mesh penetration 7 years after colpopexy. Int J Surg Case Rep 2016;24:219–22. 27289042 10.1016/j.ijscr.2016.04.047 PMC4910140

[9] Jenson MDA Scranton R Antosh DD Lumbosacral osteomyelitis and discitis with phlegmon following laparoscopic sacral colpopexy. Cureus 2016;8:e671. 27551651 10.7759/cureus.671 PMC4977220

[10] Brito LG Giraudet G Lucot JP Spondylodiscitis after sacrocolpopexy. Eur J Obstet Gynecol Reprod Biol 2015;187:72. 25758558 10.1016/j.ejogrb.2015.02.024

[11] Tymchak ZA Epp A Fourney DR Lumbosacral discitis-osteomyelitis after mesh abdominosacrocolpopexy. Spine J 2015;15:194–5. 25117721 10.1016/j.spinee.2014.08.004

[12] Vujovic Z Cuarana E Campbell KL Lumbosacral discitis following laparoscopic ventral mesh rectopexy: a rare but potentially serious complication. Tech Coloproctol 2015;19:263–5. 25720459 10.1007/s10151-015-1279-4

[13] Apostolis CA Heiselman C Sacral osteomyelitis after laparoscopic sacral colpopexy performed after a recent dental extraction: a case report. Female Pelvic Med Reconstr Surg 2014;20:e5–7. 25185618 10.1097/SPV.0000000000000092

[14] Anand M Tanouye SL Gebhart JB Vesicosacrofistulization after robotically assisted laparoscopic sacrocolpopexy. Female Pelvic Med Reconstr Surg 2014;20:180–3. 24763162 10.1097/SPV.0000000000000033

[15] Grimes CL Tan-Kim J Garfin SR Sacral colpopexy followed by refractory Candida albicans osteomyelitis and discitis requiring extensive spinal surgery. Obstet Gynecol 2012;120:464–8. 22825267 10.1097/AOG.0b013e318256989e

[16] Voelker A Hoeckel M Heyde CE Lumbosacral spondylodiscitis after sacral colpopexy of a sigmoid neovagina in a patient with vaginal melanoma. Surg Infect (Larchmt) 2012;13:134–5. 22439780 10.1089/sur.2011.083

[17] Rajamaheswari N Agarwal S Seethalakshmi K Lumbosacral spondylodiscitis: an unusual complication of abdominal sacrocolpopexy. Int Urogynecol J 2012;23:375–7. 21887545 10.1007/s00192-011-1547-4

[18] Roth TM Reight I Laparoscopic mesh explantation and drainage of sacral abscess remote from transvaginal excision of exposed sacral colpopexy mesh. Int Urogynecol J 2012;23:953–5. 22237786 10.1007/s00192-011-1630-x

[19] Draaisma WA van Eijck MM Vos J Lumbar discitis after laparoscopic ventral rectopexy for rectal prolapse. Int J Colorectal Dis 2011;26:255–6. 20532532 10.1007/s00384-010-0971-0

[20] Collins SA Tulikangas PK LaSala CA Complex sacral abscess 8 years after abdominal sacral colpopexy. Obstet Gynecol 2011;118:451–4. 21768851 10.1097/AOG.0b013e3182234e7c

[21] Muffly TM Diwadkar GB Paraiso MF Lumbosacral osteomyelitis after robot-assisted total laparoscopic hysterectomy and sacral colpopexy. Int Urogynecol J 2010;21:1569–71. 20532751 10.1007/s00192-010-1187-0

[22] Nosseir SB Kim YH Lind LR Sacral osteomyelitis after robotically assisted laparoscopic sacral colpopexy. Obstet Gynecol 2010;116: Suppl 2: 513–5. 20664437 10.1097/AOG.0b013e3181e10ea6

[23] Downing KT Vertebral osteomyelitis and epidural abscess after laparoscopic uterus-preserving cervicosacropexy. J Minim Invasive Gynecol 2008;15:370–2. 18439515 10.1016/j.jmig.2007.12.006

[24] Taylor GB Moore RD Miklos JR Osteomyelitis secondary to sacral colpopexy mesh erosion requiring laminectomy. Obstet Gynecol 2006;107:475–7. 16449155 10.1097/01.AOG.0000187949.87223.06

[25] Hart SR Weiser EB Abdominal sacral colpopexy mesh erosion resulting in a sinus tract formation and sacral abscess. Obstet Gynecol 2004;103:1037–40. 15121599 10.1097/01.AOG.0000121829.55491.0d

[26] Salman MM Hancock AL Hussein AA Lumbosacral spondylodiscitis: an unreported complication of sacrocolpopexy using mesh. BJOG 2003;110:537–8. 12742344

[27] Beloosesky Y Grinblat J Dekel A Vertebral osteomyelitis after abdominal colposacropexy. Acta Obstet Gynecol Scand 2002;81:567–8. 12047314 10.1034/j.1600-0412.2002.810617.x

[28] Kapoor B Toms A Hooper P Infective lumbar discitis following laparascopic sacrocolpopexy. J R Coll Surg Edinb 2002;47:709–10. 12463713

[29] Cosson M Narducci F Querleu D Experimental use of laparoscopic material: report of a case of spondylodiscitis after laparoscopic sacropexy with Taker. Ann Chir 2001;126:554–6. 11486539 10.1016/s0003-3944(01)00554-5

[30] Weidner AC Cundiff GW Harris RL Sacral osteomyelitis: an unusual complication of abdominal sacral colpopexy. Obstet Gynecol 1997;90:689–91. 11770599 10.1016/s0029-7844(97)00306-2

[31] Cranney A Feibel R Toye BW Osteomyelitis subsequent to abdominal-vaginal sacropexy. J Rheumatol 1994;21:1769–70. 7799366

[32] Cailleux N Daragon A Laine F Infectious spondylodiscitis after a cure for genital prolapse. 5 cases. J Gynecol Obstet Biol Reprod (Paris) 1991;20:1074–8. 1811004

